# A simple, inexpensive, robust and sensitive dot-blot assay for equal detection of the nonstructural-1 glycoprotein of all dengue virus serotypes

**DOI:** 10.1186/1743-422X-10-126

**Published:** 2013-04-22

**Authors:** Andrew KI Falconar, Claudia ME Romero-Vivas

**Affiliations:** 1 Laboratorio de Enfermedades Tropicales, Departmento de Medicina, Universidad del Norte, Km 5 Antigua via Puerto Colombia, Barranquilla, Colombia

**Keywords:** Dengue virus, Nonstructural-1 glycoprotein, Diagnostic, Monoclonal antibody, Dot-blot, Sensitivity, Low-cost

## Abstract

**Background:**

Detection of dengue virus (DENV) soluble/excreted (s/e) form of the nonstructural-1 (NS1) glycoprotein in patient acute-phase sera is ideal for diagnosis. The commercially-available detection assays are, however, too expensive for routine use and have low specificity, particularly for the s/e NS1 glycoprotein of DENV-2 and DENV-4, which are important causes of lethal human disease worldwide.

**Methods:**

Mouse monoclonal antibodies (MAbs) were generated and screened against s/e NS1 glycoprotein purified from each DENV serotype to obtain those that reacted equally with each serotype, but not with yellow fever virus (YFV) s/e NS1 glycoprotein or human serum proteins. One MAb, MAb 2C4.6, was further tested against these DENV glycoproteins in human sera using simple, peroxidase-labelled secondary antibody/substrate-developed dot-blot assays.

**Results:**

Optimal quenching of endogenous human serum peroxidases was attained using 3% H_2_O_2_ in H_2_0 for 5 min. MAb 2C4.6 showed an acceptable detection sensitivity of < 32 ng/ml for the s/e NS1 glycoprotein of each DENV serotype but did not cross-react with the YFV s/e NS1 glycoprotein or human serum proteins. By contrast, the LX1 epitope-specific MAb, 3D1.4, showed similar detection sensitivity against only the DENV-1 NS1 glycoprotein, consistent with results from commercial DENV s/e NS1 glycoprotein detection assays.

DENV s/e NS1 glycoproteins were stable in human sera after drying on the nitrocellulose membranes and storage for one month at ambient temperature (28°C) before being processed. The total assay time was reduced to 3 h without any loss of detection sensitivity. This dot-blot format was ideal for the circulating immune complex disruption step, which is required for increased DENV s/e NS1 glycoprotein detection.

**Conclusions:**

This is the first study to determine the detection sensitivity of MAbs against known concentrations of s/e NS1 glycoprotein from each DENV serotype. The preparation of patient serum samples for dot-blot assays can be performed by staff with a basic level of training and storage at low temperatures (e.g., -80°C) is not necessary. These simple, inexpensive (US$ 0.05/sample), robust, sensitive and relatively rapid assays, using improved MAbs such as MAb 2C4.6, should be ideal for the diagnosis of all DENV serotypes in DENV endemic regions.

## Background

Dengue viruses (DENVs) are the most important vector-borne human viruses in the world, causing an estimated 50–100 million infections in 100 countries and resulting in a self-limiting febrile illness called dengue fever (DF), which is sometimes associated with haemorrhage [1]. Approximately 500 000 cases result in the more severe, life-threatening forms, due to plasma leakage, severe haemorrhage, shock and organ failure called either severe dengue disease (SDD) or dengue haemorrhagic fever/dengue shock syndrome (DHF/DSS). Up to 12 500 people (2.5% of all DF cases) die from SDD (DHF/DSS) annually [1]. Four DENV serotypes (DENV-1 to DENV-4) have been identified, each of which may cause SDD (DHF/DSS).

During DENV infections, high concentrations of the native homo-hexameric form of the nonstructural-1 (NS1) glycoprotein are secreted from infected mammalian cells along with infectious DENV virions [2]. Purified secreted/extracellular (s/e) DENV-2 NS1 glycoprotein added to normal human sera could be detected by an enzyme-linked immunosorbent assay (ELISA) using a DENV NS1 glycoprotein complex-reactive monoclonal antibody, (MAb) 3D1.4 [3-5], with a sensitivity of 15 ng/ml [6]; although the DENV-2 NS1 glycoprotein-specific MAb, 1H7.4 [3], was slightly more sensitive (4 ng/ml) [6]. However, the ELISA using MAb 1H7.4 failed to detect the s/e NS1 glycoprotein in acute-phase sera from patients with primary DENV-2 infections collected in Thailand [6]. This assay also only detected the s/e NS1 glycoprotein in less than 50% (DF: 40% and DHF/DSS: 45%) of acute-phase sera from patients with secondary DENV-2 infections, at concentrations from 70 ng/ml up to 15,000 ng/ml [6]. Since these serum samples had undergone several freeze-thaw cycles, the results demonstrated the lability of the DENV s/e NS1 glycoprotein, probably through disruption of the relevant LD2 epitope (amino acid 25–33), [4] or loss of the LD2 epitope through cleavage of the NS1 glycoprotein at the conserved (amino acid 100–GKRS–103) dibasic amino-terminal protease cleavage site [7]. However, a capture ELISA using mouse and rabbit polyclonal antibodies (PAbs) detected the s/e NS1 glycoprotein in a moderate proportion of acute-phase sera from patients with either primary (77% (10/13): 40 to 2,000 ng/ml) or secondary (88% (14/16): 10 to 2,000 ng/ml) DENV-1 infections in French Guiana [8], identifying the s/e NS1 glycoprotein as a suitable target for DENV diagnostics.

MAbs used in DENV NS1 glycoprotein detection assays should react equally with the NS1 glycoproteins of each DENV serotype. However, MAb 3D1.4 and other MAbs (MAbs 1A12.3, 4H3.4 and 3A5.4) that bound the DENV complex LX1 epitope (amino acid 112–KYSWKTWGKA–121) on the DENV NS1 glycoproteins reacted unequally with synthetic peptides containing the corresponding LX1 epitope of each DENV serotype, as follows: DENV-1 > DENV-3 > DENV-2 = DENV-4 [4].

Subsequently available commercial DENV s/e NS1 glycoprotein detection ELISAs used MAb 3D1.4 or other likely LX1 epitope-specific MAbs (Pan-E, PanBio/Inverness, Brisbane, Queensland, Australia; Platelia, Bio-Rad Laboratories, Marnes La Coquette, France, or Standard Diagnostics Inc., Kyonggi-do, South Korea). These assays are too expensive ($US 5 to 10/sample) for routine screening of DENV-infected patients in poor, endemic areas, and have low sensitivity for the s/e NS1 glycoprotein of one or more DENV serotypes [9-11]. The Pan-E, Platelia and Standard Diagnostics DENV s/e NS1 glycoprotein detection ELISAs show low to moderate overall sensitivities with acute phase DENV serum samples (Standard Diagnostics: 55%; Platelia: 56.5% [11]; Pan-E: 56.3%; Platelia: 68.0% [10]; Pan-E: 64.9%; Platelia: 83.2% [9]). These assays also showed the highest sensitivity for DENV-1 (Standard Diagnostics: 70%; Platelia: 70% [11]; Pan-E: 79%; Platelia: 87% [10]; Pan-E 78.6%; Platelia: 93% [9]) and the lowest sensitivity for either DENV-2 (Standard Diagnostics: 41%; Platelia: 38% [11]; Pan-E: 62%; Platelia: 63% [10]; Pan-E: 76%; Platelia 82% [9]), or DENV-4 (Standard Diagnostics: 40%; Platelia 73% [11]; Pan-E: 52%; Platelia: 79% [10]; Pan-E: 36%; Platelia: 71% [9]). These results accounted for a large multi-country evaluation giving the lowest sensitivities of Pan-E and Platelia s/e NS1 detection ELISAs with acute-phase sera from Nicaraguan patients (Pan-E: 30%; Platelia: 37%), due to nearly all being infected with DENV-2 (94%), and not with DENV-1 (6%) or DENV-3 (0%) [10]. Thus, in summary, the strength of these assay reactions varied with DENV serotype, in the following order: DENV-1 > DENV-3 > DENV-2 > DENV-4, similar to the anti-LX1 epitope MAbs [4].

More recent commercial DENV NS1 glycoprotein detection assays had even lower sensitivities. This was demonstrated in two well-conducted studies of NS1 antigen strip assays, Pan-E (average sensitivity: 45% (range 30–59%), PanBio (average sensitivity: 58.6%: range 48.2–68.4%), and Bio-Rad (average sensitivity: 58.6%: range 48.2–68.4%) and the Standard Diagnostics lateral flow s/e NS1 detection assay (mean sensitivity: 48.5%: range 38.5–58.7%) [11,12].

Commercially-available DENV NS1 glycoprotein detection/semi-quantification assays are too expensive for routine use in nearly all DENV endemic regions of the world, and all show significant differences in ability to detect the s/e NS1 glycoprotein of each DENV serotype [9-11,13-16]. Therefore there is an urgent need to develop improved MAbs for use in inexpensive DENV s/e NS1 glycoprotein detection assays.

We previously showed that the s/e NS1 glycoprotein from all four DENV serotypes could be detected in Indonesian patient sera using a simple dot-blot format, particularly when acid treatment and neutralisation steps were used [17]. However, these assays used an electrochemiluminescence (ECL) substrate coupled with photographic film, which is too expensive for routine use in DENV endemic areas. One advantage was that the glycoproteins were stable for month-long periods when dot-blotted, blocked, and dried onto nitrocellulose membranes before being developed [Falconar, unpublished]. It is also likely that improved immunoperoxidase detection sensitivity could be obtained in Western blot or dot-blot assays by incorporating Ni^2+^ ions into the 3,3’diaminobenzidine substrate, as demonstrated in immunohistochemical studies [18].

In the present study, we aimed to determine: a) whether we could generate mouse MAbs that could more equally detect the s/e NS1 glycoprotein of each serotype without cross-reacting with yellow fever virus (YFV) s/e NS1 glycoprotein or human serum proteins; b) the optimal parameters for quenching endogenous human serum peroxidases in simple dot-blot assays using one of these MAbs and standard 4-chloro-1-naphthol and 3,3’-diaminobenzidine tetrahydrochloride (CND) substrate precipitation; c) the detection sensitivities of such a MAb against the purified s/e NS1 glycoprotein of each DENV NS1 glycoprotein in these dot-blot assays; d) whether such a MAb would show superior detection of the s/e NS1 glycoprotein of each DENV serotype compared with MAb 3D1.4, which bound the LX1 epitope; e) whether the detection sensitivity could be increased by incorporating 1 mM Ni^2+^ ions into the CND substrate step; f) whether the DENV s/e NS1 glycoproteins in human sera were stable when blotted onto the membranes, blocked, dried and stored at either ambient temperature (28°C) or at 4°C, -20°C or -80°C before being processed; g) whether these assays could be performed within a moderate time period (e.g. 3 hours); and h) the approximate cost of these assays (US$/sample).

## Results

### Purification of s/e NS1 glycoproteins of each DENV serotype and YFV

Large (250–500 μg) quantities of the soluble/extracellular (s/e) NS1 glycoproteins were obtained from DENV-1 (S#14 strain), DENV-2 (S#42 strain), DENV-3 (S#25strain) and DENV-4 (S#10 strain) serotypes isolated in our study site [19,20] and from yellow fever virus (YFV) (17D-204 strain) by immunoaffinity purification from large infected Vero cell cultures as described previously [7,21]. These purified s/e NS1 glycoproteins were used for all mouse immunisations and immunoassays.

### Monoclonal antibodies (MAbs)

**Figure 1:**
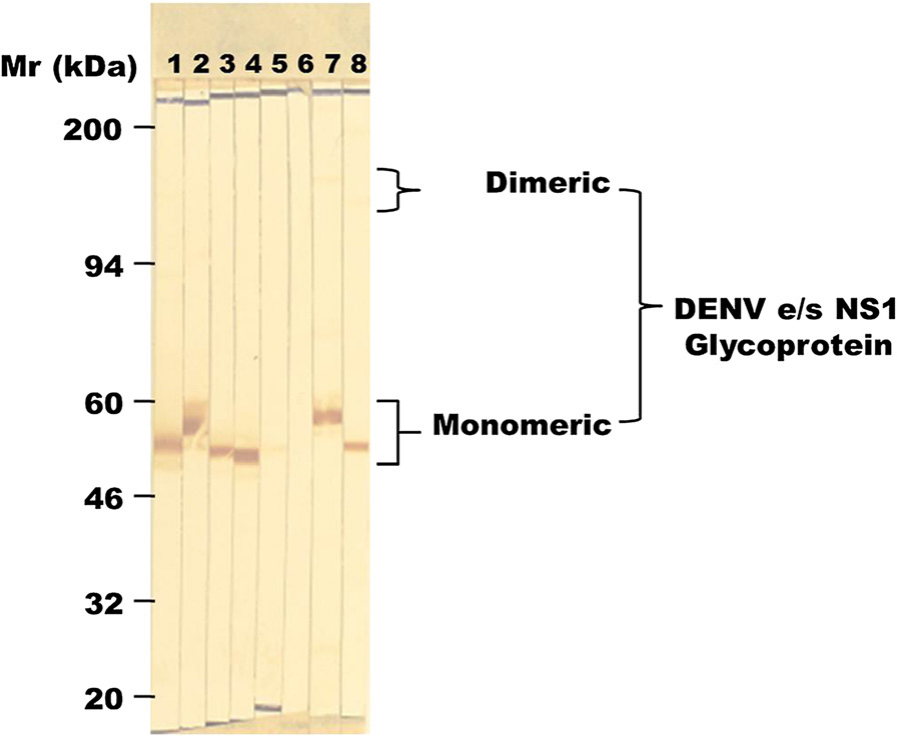
**MAb 2C4.6 reactions with the s/e NS1 glycoprotein of each DENV serotype in Western blot assays.** Preparative Western blot strips containing approximately 35 ng bands of the secreted/extracellular (s/e) NS1 glycoprotein of DENV-1 (strip 1), DENV-2 (strip 2), DENV-3 (strip 3), DENV-4 (strip 4), 2-mercaptoethanol-reduced DENV-2 (strip 5), or purified YFV (strip 6), a crude lysate of DENV-2 infected Vero (monkey kidney fibroblast) cells (strip 7), or a human serum sample previously treated with 3% (vol/vol) H_2_O_2_/PBS for 60 min to quench endogenous human serum peroxidases before addition of SDS-stacking buffer and the purified DENV-2 s/e NS1 glycoprotein (strip 8). The strips were taped together and simultaneously processed by sequential reaction steps with MAb 2C4.6, peroxidase-labelled goat anti-mouse IgG (H&L) and 4-chloro-1-naphthol/3,3’diaminobenzidine (CND) substrate. Due to variations in the heights of the preparative strips of each sample, the locations of the average molecular weight (Mr) markers (20 to 200 kDa), and the approximate Mr ranges where the dimeric and monomeric forms of DENV s/e NS1 glycoproteins, are shown. The DENV s/e NS1 glycoproteins were almost exclusively converted into their monomeric forms by heat (100°C for 3 min) treatment.

In this study, only low numbers of the MAb-producing hybridomas (1.7%: 16/960) showed strong cross-reactions with the s/e NS1 glycoprotein of multiple different DENV serotypes. MAb 2C4.6 was identified as a suitable candidate for further studies by its reactions in the preparative Western blot strip assays (Figure 1). In this study, supernatant from two 48-well plates containing MAb 2C4.6 (1 ml volume diluted 1:2 in PBS/T/M) reacted strongly with the bands containing 35 ng of the purified s/e NS1 glycoprotein of each DENV serotype but reacted very weakly with the DENV-2 NS1 glycoprotein when it was reduced with 2-mercaptoethanol. It did not cross-react with either the s/e NS1 glycoprotein of YFV, human serum proteins, or other cellular or DENV-2 proteins in a DENV-2-infected Vero cell lysate. The DENV s/e NS1 glycoproteins were almost exclusively converted into their monomeric forms after heat treatment. Therefore, MAb 2C4.6, which is of the immunoglobulin G (IgG) 2b subclass, specifically bound to a conformationally-dependent (2-mercaptoethanol reduction-sensitive) epitope on the s/e NS1 glycoprotein of each DENV serotype.

### Optimal endogenous human serum peroxidase quenching for DENV-2 s/e NS1 glycoprotein detection by MAb 2C4.6 in dot-blot assays

**Figure 2:**
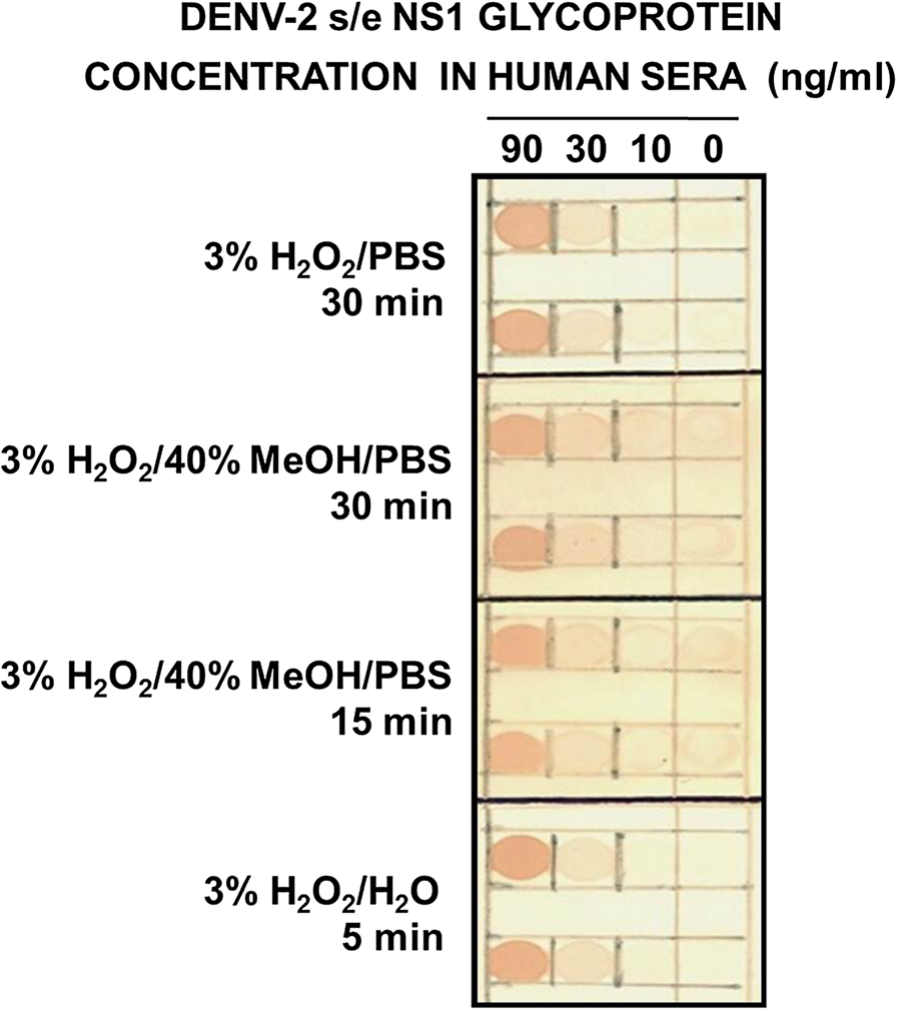
**Optimal quenching of endogenous human serum peroxidases in the DENV s/e NS1 glycoprotein dot-blot detection assay.** Serial three-fold dilutions (90, 30 and 10 ng/ml) of immunoaffinity-purified DENV-2 s/e NS1 glycoprotein, and buffer without DENV-2 NS1 glycoprotein (0 ng/ml), prepared in undiluted human sera were added at 10 μl volumes to four sets of paired 5-mm squares marked on nitrocellulose membranes. These membranes were then dried and cut for reaction with each of the different solutions trialled for quenching endogenous human serum peroxidases (as shown), before being washed and blocked. These membranes were then processed by sequential reaction steps with MAb 2C4.6, peroxidase-labelled goat anti-mouse IgG (H&L) and 4-chloro-1-naphthol/3,3’diaminobenzidine (CND) substrate. The results were obtained from one of duplicate experiments performed that gave similar results.

**Figure 3:**
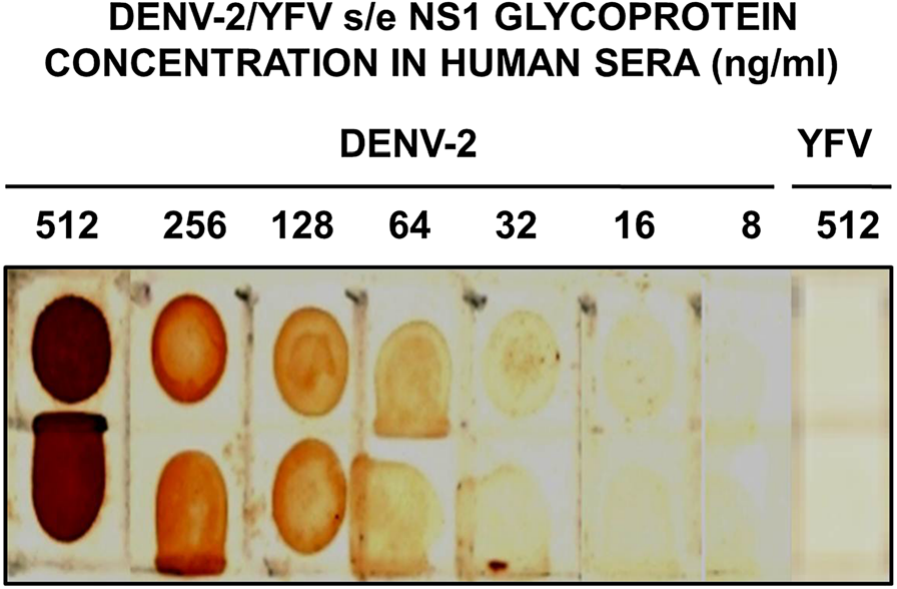
**Maximum dot-blot detection sensitivity of MAb 2C4.6 against the DENV-2 s/e NS1 glycoprotein.** Serial two-fold dilutions (512 to 8 ng/ml) of immunoaffinity-purified DENV-2 s/e NS1 and YFV s/e NS1 (512 ng/ml) glycoproteins prepared in undiluted human sera were added at 10 μl volumes to paired 5-mm squares marked on a nitrocellulose membrane. After being dried and treated with 3% H_2_O_2_/H_2_O for 5 min to quench the endogenous human serum peroxidases, this membrane was washed and blocked. The membrane was then processed by sequential reaction steps with MAb 2C4.6, peroxidase-labelled goat anti-mouse IgG (H&L) and 4-chloro-1-naphthol/3,3’diaminobenzidine (CND) substrate. The results were obtained from one of duplicate experiments performed that gave similar results.

The ability of 3% H_2_O_2_ in either PBS, 40% MeOH/PBS or H_2_O to reduce the background noise in the dot-blot assays was assessed using three-fold serial dilutions of DENV-2 s/e NS1 glycoprotein prepared in neat human sera (Figure 2). The blots treated with 3% H_2_O_2_ in H_2_O for 5 min gave slightly lower backgrounds than treatment with 3% H_2_O_2_ in PBS for 30 min. By contrast, the use of 3% H_2_O_2_ diluted in 40% MeOH/PBS for 15 or 30 min resulted in higher backgrounds, thereby reducing the detection sensitivity. The apparent maximum optimal detection sensitivity for the DENV-2 s/e NS1 glycoprotein was approximately 30 ng/ml (0.3 ng/dot). The dot-blot assays were therefore apparently more sensitive than Western blot assays; in previous studies using other anti-DENV NS1 glycoprotein-specific MAbs, the maximum sensitivity was obtained with a 10 ng band [7,21]. Using the optimal 3% H_2_O_2_ in H_2_O for 5 min quenching method, with two-fold serial dilutions, the maximum detection sensitivity for the s/e DENV-2 NS1 glycoprotein was < 32 ng/ml using MAb 2C4.6 (Figure 3). An attempt to improve the sensitivity by the addition of 1 mM Ni^2+^ to the 4-chloro-1-naphthol/3, 3’diaminobenzidine substrate mixture resulted in reduced DENV s/e NS1 glycoprotein detection sensitivities due to precipitation of the 4-chloro-1-naphthol (data not shown).

Consistent with the inability of MAb 2C4.6 to react with a 35 ng band of the yellow fever virus s/e NS1 glycoprotein in the Western blot assay (Figure 1), this MAb also showed no detectable reaction with 5.12 ng (512 ng/ml) concentration of this glycoprotein in these dot-blot assays (Figure 3).

### Comparison of the reaction of MAb 2C4.6 and 3D1.4 against the s/e NS1 glycoprotein of all four DENV serotypes in dot-blot assays

**Figure 4:**
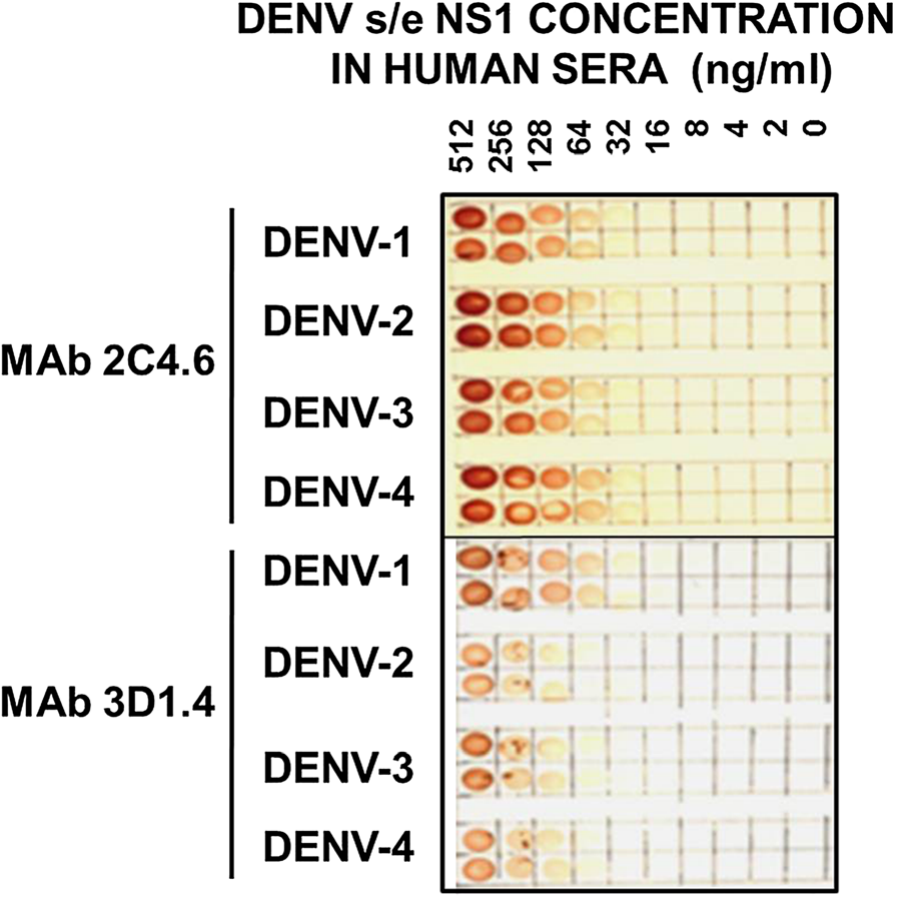
**Detection sensitivities of MAb 2C4.6 and MAb 3D1.4 against the s/e NS1 glycoprotein of each DENV serotype.** Serial two-fold dilutions from 512 to 2 ng/ml of immunoaffinity-purified s/e NS1 glycoprotein of each DENV serotype (DENV-1 to DENV-4) or buffer only (0), prepared in undiluted human sera were added at 10 μl volumes to duplicate paired 5-mm squares marked on a nitrocellulose membrane. After being dried and treated with 3% H_2_O_2_/H_2_O for 5 min to quench the endogenous human serum peroxidases, this membrane was washed and blocked. After being washed and dried, the membrane was cut into two equal pieces and then processed by sequential reaction steps with either MAb 2C4.6 or MAb 3D1.4, peroxidase-labelled goat anti-mouse IgG (H&L) and 4-chloro-1-naphthol/3,3’diaminobenzidine (CND) substrate. The results were obtained from one of duplicate experiments performed that gave similar results.

The ability of MAb 3D1.4 to react with the s/e NS1 glycoprotein of each DENV serotype in the dot-blot assays was compared to that of MAb 2C4.6. MAb 3D1.4 bound more strongly to the corresponding LX1 epitope synthetic peptide sequence of the DENV-1 NS1 glycoprotein than the corresponding peptide sequences of the other DENV serotype [4], and the DENV-1 NS1 glycoprotein using the Pan-E ELISA on serum samples from DENV-1 infected patients [9,10]. In this study, MAb 3D1.4 showed a similar detection sensitivity to MAb 2C4.6 (< 32 ng/ml) against the DENV-1 s/e NS1 glycoprotein, but had lower detection sensitivities against the s/e NS1 glycoprotein of DENV-3 (approximately 64 ng/ml), DENV-4 (approximately 100 ng/ml), and DENV-2 (approximately 128 ng/ml) (Figure 4). Thus, MAb 2C4.6 showed superior detection sensitivity to the s/e NS1 glycoprotein of each DENV serotype than MAb 3D1.4 in these assays.

MAbs 1G5.4-A1-C3 and 1B6.4d, of the IgG2b subclass, also generated against the DENV NS1 glycoprotein showed the same DENV-2 > DENV-4 > DENV-1 > DENV-3 and DENV-2-specific reactions in these dot-blot assays (data not shown) as were observed in Western blot assays, respectively [3,21,22].

### Reaction of MAb 2C4.6 with s/e NS1 glycoprotein of each DENV serotype and their stability when dried on nitrocellulose membranes and stored at different temperatures

Serial two-fold dilutions (from 512 to 2 ng/ml) of the purified s/e NS1 glycoprotein of each DENV serotype were prepared in human sera and then added at 10 μl/dot to nitrocellulose membranes. The membranes were dried, quenched for 5 min with 3% H_2_O_2_/H_2_O, blocked, washed with PBS containing 0.02% NaN_3_, and then air dried. After processing, MAb 2C4.6 was observed to bind equally to the s/e NS1 glycoprotein of each DENV serotype, again with a maximum detection sensitivity of < 32 ng/ml (< 0.32 ng dot) against each DENV serotype.

**Figure 5:**
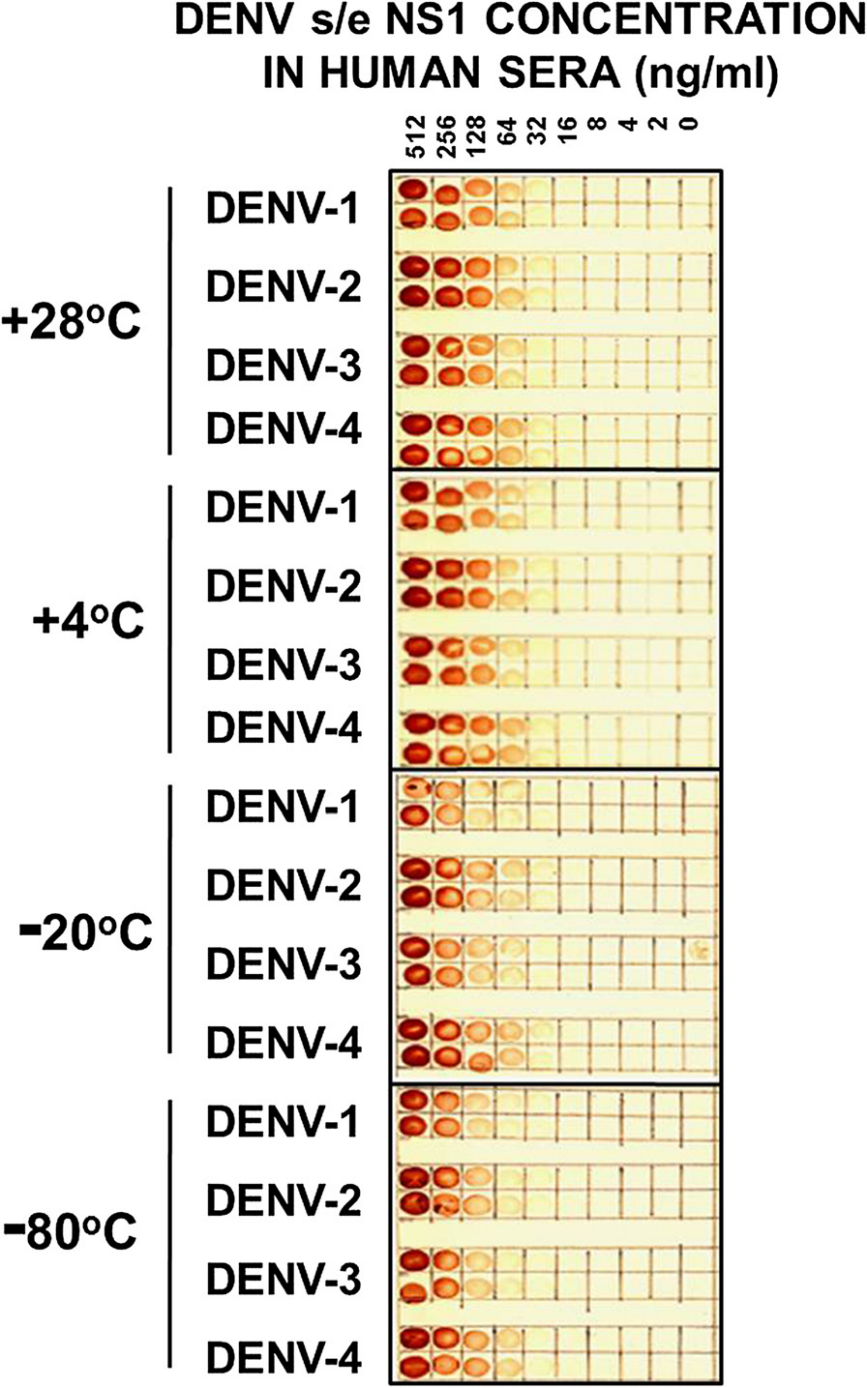
**Detection sensitivities of MAb 2C4.6 against DENV s/e NS1 glycoproteins stored at different temperatures.** Serial two-fold dilutions from 512 to 2 ng/ml of immunoaffinity-purified s/e NS1 glycoprotein of each DENV serotype (DENV-1 to DENV-4) or buffer only (0), prepared in undiluted human sera were added at 10 μl volumes to four paired 5-mm squares marked on a nitrocellulose membrane. After being dried and treated with 3% H_2_O_2_/H_2_O for 5 min to quench the endogenous human serum peroxidases, this membrane was washed and blocked. After being washed and dried, the membrane was cut into four equal pieces, sealed in plastic bags and stored either at ambient temperature (mean 28°C), 4°C, -20°C or -80°C for 1 month. These membranes were then processed by sequential reaction steps with MAb 2C4.6, peroxidase-labelled goat anti-mouse IgG (H&L) and 4-chloro-1-naphthol/3,3’diaminobenzidine (CND) substrate. The results were obtained from one of duplicate experiments performed that gave similar results.

We previously observed that s/e DENV NS1 glycoproteins were stable when dried on nitrocellulose membranes in dot-blot and Western blot assay formats at ambient temperatures for month-long periods (Falconar, unpublished). The dot-blot assays developed in the current study were also assessed for their stability. The s/e NS1 glycoprotein of each DENV serotype diluted in human sera were quenched for 5 min with 3% H_2_O_2_/H_2_O, blocked, washed with PBS containing 0.02% NaN_3_, air dried, placed in sealed plastic bags and stored for 1 month at ambient temperature (mean: 28°C), 4°C, -20°C or -80°C before processing. After processing them, the detection sensitivity for the NS1 glycoprotein of each DENV serotype remained at < 32 ng/ml for the membranes stored at 28°C and 4°C (Figure 5). However, the detection sensitivity for one or more DENV serotypes was reduced for membranes stored at -20°C and -80°C.

### Reduction of the total dot-blot assay time to 3 hours

**Figure 6:**
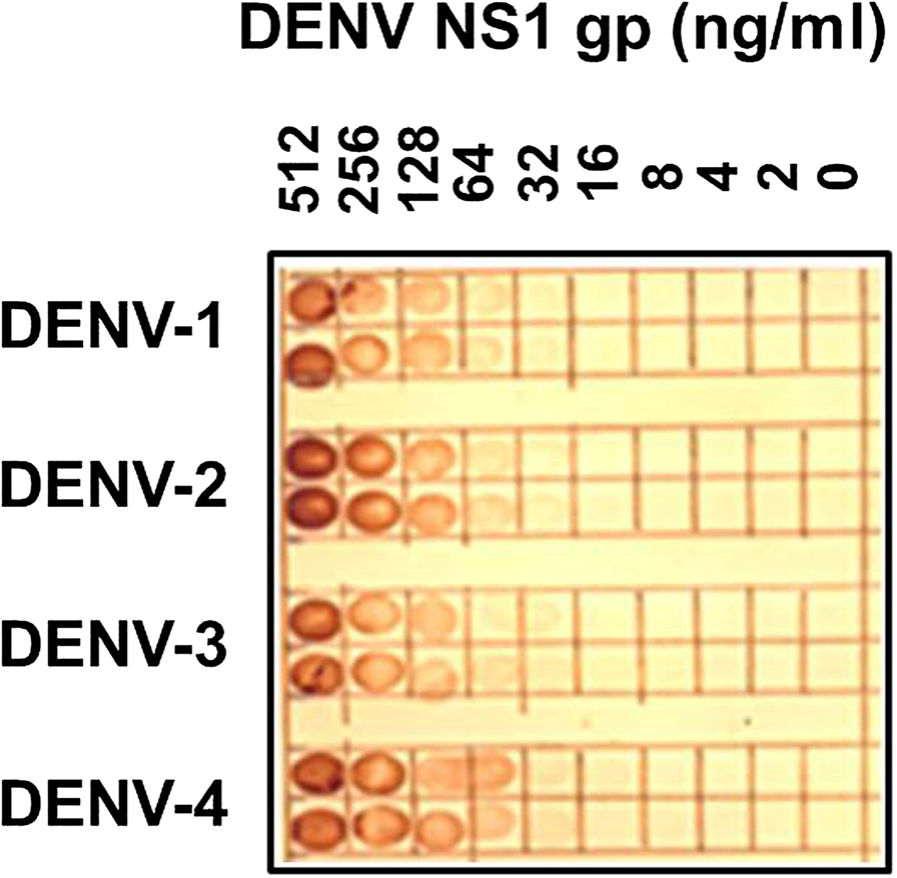
**Detection sensitivities of MAb 2C4.6 against the DENV s/e NS1 glycoproteins in a 3 h assay.** The maximum detection sensitivity of the assay was assessed against the same serial dilutions of immunoaffinity-purified s/e NS1 glycoproteins when the total dot-blot assay time was reduced to 3 h. For this assay, serial two-fold dilutions from 512 to 2 ng/ml of immunoaffinity-purified s/e NS1 glycoprotein of each DENV serotype (DENV-1 to DENV-4) or buffer only (0), prepared in undiluted human sera were added at 10 μl volumes to paired 5-mm squares marked on a nitrocellulose membrane. After being dried and treated with 3% H_2_O_2_/H_2_O for 5 min to quench the endogenous human serum peroxidases, this membrane was washed and blocked before being processed by sequential reaction steps with MAb 2C4.6, peroxidase-labelled goat anti-mouse IgG (H&L) and 4-chloro-1-naphthol/3,3’diaminobenzidine (CND) substrate. The results were obtained from one of duplicate experiments performed that gave similar results.

To determine if the dot-blot assay could be performed in 3 h, the same serial dilutions of the s/e NS1 glycoprotein of all four DENV serotypes were used, and the following steps were performed at ambient temperature (28°C): i) loading and drying (40 min), ii) quenching (5 min), iii) washing (0.3 min), iv) blocking (50 min), iv) washing (0.3 min), v) primary MAb (MAb 2C4.6) reaction (30 min), vi) washing (3 × 1 min), vii) labelled secondary antibody reaction (25 min), viii) washing (3 × 1 min), ix) substrate reaction (10 min), and x) washing (1 min) (total assay time: 178 min). In this study, there was no observable reduction in the maximum DENV-1 to -4 s/e NS1 glycoprotein detection sensitivities of < 32 ng/ml (Figure 6) compared to those obtained using the longer assay time (Figures 2, 3 and 4); therefore, these simple dot-blot assays could be performed relatively rapidly.

## Discussion

### Reaction bias of the MAbs used to detect the DENV s/e NS1 glycoprotein

We previously obtained five MAbs (1A12.3, 3D1.4, 3A5.4, 4H3.4 and 1C6.3), which were used to define DENV complex epitopes on the NS1 glycoprotein [3], and another MAb was generated as a sub-clone (1G5.4-A1-C3) [21]. These MAbs either did not react equally with the s/e NS1 glycoproteins of each DENV serotype in Western blot assays (e.g., MAbs 1C6.3 and 1G5.4-A1-C3) [5,21,22] or with the corresponding LX1 epitope sequences of each DENV serotype prepared as synthetic peptides (MAbs 3D1.4, 3A5.4, 4H3.4 and 1A12.3) [4]. The present study confirmed that MAb 3D1.4 did not react equally with the s/e NS1 glycoprotein from the different DENV serotypes, showing high sensitivity for the s/e NS1 glycoprotein of DENV-1 (< 32 ng/ml), but lower sensitivity for those of the other serotypes, particularly DENV-2 (< 128 ng/ml) (Figure 4). It is therefore not surprising that the commercially-available DENV s/e NS1 glycoprotein detection assays showed much higher sensitivities with DENV-1 infected patients’ sera than with sera from patients infected with the other DENV serotypes, particularly DENV-2 and DENV-4 [9-11,14]. This suggests that these assays all employ MAbs binding to the LX1 epitope. We attempted to generate better MAbs by immunising BALB/c mice with a mixture of s/e NS1 glycoproteins from each DENV serotype, screening resultant hybridomas against known concentrations of these glycoproteins using ELISAs, preparative Western blot strips, and dot-blot assays.

### Sensitivity of the DENV s/e NS1 glycoprotein assays

Our simple dot-blot assays showed maximum sensitivities of < 32 ng/ml, more than adequate to detect concentrations of s/e NS1 glycoproteins from DENV-1 and DENV-3 (from 43 to 15 219 ng/ml) that were detected in patient sera using the Platelia ELISA [15]. In contrast, the Platelia assay could only detect low concentrations (2–163 ng/ml) of the s/e NS1 glycoprotein in sera from patients infected with DENV-2. Indeed, the DENV-2 s/e NS1 glycoprotein was undetectable in one-third of these patients, despite levels of viremia similar to those infected with DENV-1 and DENV-3 [15]. These observations indicate that the DENV-2 s/e NS1 glycoprotein was poorly detected by the MAb used in this assay. This was strongly supported by detection of DENV-2 s/e NS1 glycoprotein in 71% (10/14) and 89% (16/18) of patients who subsequently developed DF and DHF, respectively, and their high concentrations of the s/e NS1 glycoprotein (up to 14 000 ng/ml) and high virus titres (viraemias) [23], similar to those in DENV-1 and DENV-3 infected patients [15]. In contrast to the Platelia assay, this non-commercially developed capture ELISA incorporated a DENV-2 NS1 glycoprotein-specific MAb, 1H7, along with rabbit anti-DENV-2 NS1 glycoprotein PAbs [3,6,23]. Importantly, this non-commercial assay detected the DENV-2 s/e NS1 glycoprotein at > 600 ng/ml in 21% (3/14) and 72% (13/18) of the patients who subsequently developed DF and DHF, respectively, thereby providing a sensitivity of 72% and a specificity of 79% for identifying patients who subsequently developed SDD (DHF/DSS) [23]. However, unlike the DENV-2 NS1 glycoprotein specific MAb 1H7.4 and the MAbs used in commercial DENV s/e NS1 glycoprotein detection assays, MAb 2C4.6 reacted equally with the s/e NS1 glycoproteins of each DENV serotype in human serum samples.

### Effect of patient PAbs on DENV s/e NS1 glycoprotein detection assays

Both the Platelia and Pan-E detection ELISAs showed markedly reduced sensitivity when used to test sera from patients with secondary DENV-2 infections compared with sera from patients with primary DENV-2 infections [24]. Furthermore, the presence of anti-DENV IgM and, to a much greater extent, anti-DENV IgG PAbs, in sera from Vietnamese patients reduced the sensitivity of the Platelia assay [13]. Thus, the combination of MAbs that do not react equally with the s/e NS1 glycoproteins of each DENV serotype and the presence of patient PAbs against the DENV NS1 glycoproteins (generated during secondary DENV infections) is likely to be responsible for the low sensitivity of these commercial assays. The incorporation of simple acid treatment and neutralisation steps, which disrupt circulating immune complexes (CICs) present in the patient sera, improved the detection of the DENV s/e NS1 glycoprotein (particularly in patients infected with DENV-2) in dot-blot assays of acute-phase primary and secondary sera from Indonesian patients [17]. While these acid treatment and neutralisation steps had little effect on the sensitivity to the DENV-1, DENV-3 and DENV-4 s/e NS1 glycoproteins (although only one DENV-4-infected patient was tested), they markedly increased the detection of the DENV-2 s/e NS1 glycoprotein in infected patient sera from 25% to 100% [17]. In contrast, only a modest increase in sensitivity (9%) was reported when these acid treatment and neutralisation steps were performed on patient sera prior to testing in the Platelia assay; however, the DENV serotypes in those patients had not been determined [25]. The weak reaction between MAb 3D1.4 and the s/e NS1 glycoprotein of DENV-4 in the dot-blot assays observed in the current study (Figure 4) was consistent with the lower detection rates observed when the commercial assays were used to test acute-phase sera from patients infected with DENV-4 [9-11,14]. Further studies are needed to assess whether these acid treatment and neutralisation steps are more suited to improving the detection of the DENV-2 and DENV-4 s/e NS1 glycoproteins present in patient CICs in the dot-blot assays, as they bind more rapidly to nitrocellulose membranes after neutralisation than they do in the capture ELISAs where such delays may result in the reformation of CICs. Acid treatment of patient sera for the DENV s/e NS1 glycoprotein detection dot-blot assays was however performed for 60 minutes before neutralization [17], thereby adding additional time to the assay. This would have also dissociated the DENV NS1 glycoproteins into their monomeric forms [7]. Alternative agents that disrupt patient CICs more quickly are likely to be useful in these assays (see below).

### Combination of single-patient DENV-specific IgM/IgG and DENV s/e NS1 glycoprotein detection results for DENV diagnosis

The sensitivity of commercial assays is reportedly increased by combining results from DENV NS1 glycoprotein assays with those from anti-DENV-specific pre-membrane (prM) and envelope (E) (prM/E) glycoprotein IgG and/or IgM detection assays [10-12,26]. However, false-positives have been reported using some DENV NS1 glycoprotein or anti-DENV prM/E glycoprotein IgG or IgM antibody detection assays during secondary DENV infections or with sera from patients carrying other infectious agents or from healthy donors [10-12]. These false-positives may account for the reported increased sensitivity of such combined assays, in addition to the use of sera from patients predominantly infected with DENV (DENV-1 or DENV-3) serotypes, more easily detected by these commercial assays: a) DENV-1 (DENV-1: 56.3%; DENV-2: 37.6%; DENV-3: 6.6%; DENV-4: 0.0%) [26]; b) DENV-1 plus undetermined DENV serotypes (DENV-1: 39.3%; DENV-2: 16.3%; DENV-3: 6.3%; DENV-4: 8.4%; undetermined DENV serotypes: 29.7%) [11]; or c) DENV-3 plus undetermined DENV serotypes (DENV-1: 1%; DENV-2: 16.2%; DENV-3: 47.5%; DENV-4: 2.0%; undetermined: 33.3%) [12]. Importantly, some DENV patients (termed *LS patients) generated low levels of anti-DENV prM/E glycoprotein reactive IgG antibodies, even during secondary DENV infections [19]. Indeed, 40% of patient sera may be negative for IgG and IgM antibodies against the DENV E and prM glycoproteins on day 3 after the onset of fever [19], and DENV prM/E glycoprotein-specific IgM antibodies were not generated during secondary DENV infections in patients from either Asia (28% IgM-negative) [27] or the Americas (33% IgM-negative) [19]. These problems were further confirmed by the very low sensitivity observed for a commercially-available combined DENV diagnostic assay used to test a panel of sera from patients with fully characterised DENV infections, primarily secondary DENV-2 or DENV-4 infections [Falconar and Romero-Vivas, unpublished]. DENV-2 and DENV-4 are the dominant DENV serotypes in our DHF/DSS-endemic study site in the Americas (Colombia) [19,20]. These serotypes were maintained in the Americas after their introduction [28,29] and are important causes of DENV epidemics (e.g. in Mexico [30], Nicaragua [31], Guatemala and Honduras [32], Brazil [33]) and the Caribbean islands (e.g., Martinique [34]), as well as in Asia (e.g., Thailand [35], Singapore [36] and China [37]). Thus, there is an urgent need to develop simple, inexpensive, sensitive and robust NS1 glycoprotein detection assays that can equally detect the s/e NS1 glycoprotein of each DENV serotype.

### Advantages of DENV s/e NS1 glycoprotein detection dot-blot assays using improved MAbs

The failure to maintain DENV patient sera at low temperatures before testing resulted in an inability to detect the glycoprotein in acute-phase sera from patients with primary DENV-2 infections [6]. Thus, the ability to store antigenically stable prepared membranes at 28°C (Figure 5), is highly desirable. These membranes can then be immediately processed using basic equipment and reagents, and by staff with minimal training. Alternatively, they can be transferred to other laboratories at ambient temperatures for processing. As a result, large numbers of samples from patients with febrile illnesses can be screened easily, inexpensively (approximately US$ 0.05/sample, i.e., 100–200 times less expensive than commercial detection assays) and relatively rapidly for DENV infections in all endemic areas. Importantly, simple definitive clinical criteria (conjunctival injection, abdominal pain and venipuncture bleeding) can also be applied to patients by staff from such clinics with minimal training, to identify the relatively low numbers of DENV-infected patients who will subsequently develop SDD (DHF/DSS), so that prompt hospital-based therapy can be provided [19].

## Conclusions

This is the first study to assess quantitatively the sensitivity of a DENV NS1 glycoprotein detection assay using known concentrations of s/e NS1 glycoprotein from each DENV serotype. The main findings were that: i) hybridomas were generated and successfully screened for MAbs that showed an equally strong reaction with purified s/e NS1 glycoprotein of each DENV serotype; ii) optimal quenching of endogenous peroxidases in human sera was achieved using 3% H_2_O_2_ (in H_2_O) for 5 min; iii) the maximum sensitivity of MAb 2C4.6 for the s/e NS1 glycoprotein of each DENV serotype was < 32 ng/ml (< 0.32 ng/dot); iv) the addition of 1 mM Ni^2+^ to the substrate mixture reduced the sensitivity of the dot-blot assays; v) s/e NS1 glycoproteins of each DENV serotype in human sera were stable when dried on nitrocellulose membranes at ambient temperature (28°C) or when stored at 4°C for at least 1 month before processing; vi) the total assay time was reduced to 3 h without any loss of sensitivity; vii) the estimated cost of these assays was US$ 0.05/sample, and vii) the sensitivity of MAb 2C4.6 for the s/e NS1 glycoprotein of the DENV-2, DENV-3 and DENV-4 serotypes was superior to that of MAb 3D1.4 in these assays.

Dot-blot assays, such as the one developed here, have the potential for use as simple, robust, inexpensive and relatively rapid diagnostic tools in DENV-endemic countries, where there are an estimated 50-100 million infections annually [1].

### Further studies

We are presently testing whether improved alternative methods for disruption of patient CICs together with alternative detection methods can be used to increase the sensitivities of these dot-blot assays by 10 to 100-fold and to reduce the total assay time to the same (or less) than that required to perform the Pan-E, Platelia and Standard Diagnostic DENV NS1 glycoprotein detection ELISAs (2.5 hours).

We also wish to fully evaluate these dot-blot assays using large numbers of patient sera collected during the acute phase of primary and secondary DENV infections caused by each DENV serotype (i.e. n > 200) which have been typed by full IgM/IgG ELISA titrations and DENV isolation followed by serotype determinations, together with full clinical details [19,20]. Since some commercial DENV s/e NS1 detection assays have also been shown to give false positives using normal human sera, and sera from patients with some other viral or bacterial diseases [11], we will also determine whether MAb 2C4.6 will not generate such false positive reactions in these assays. If these studies are successful, MAb 2C4.6 may be suitable for use in a rapid (5-10 min) point-of-care format.

## Materials and Methods

### Ethics statement

The collection of 5 ml blood samples from two clinically healthy human volunteers to obtain the normal human sera used in this study was approved by the University del Norte Ethics Committee. All animal experiments were approved by the Universidad del Norte Ethics Committee following the guidelines for the care and use of experiment animals established by the Colombian government, and following NIH guidelines.

### Study site

Barranquilla is the principal seaport of Colombia and lies at the mouth of the Magdalena River on the Caribbean coast. All four DENV serotypes have become endemic in the city, with severe dengue disease (SDD: DHF/DSS) being confirmed since 1997. The isolation of all four DENV serotypes of DENV (DENV-1 (S#14 strain), DENV-2 (S#42 strain), DENV-3 (S#25 strain) and DENV-4 (S#10 strain) in insect (C6/36) cells was performed as described previously [19,20].

### Purification of MAbs and preparation of immunoaffinity columns

The purification of mouse MAbs of the IgG1, IgG2a and IgG2b subclasses and the preparation of immunoaffinity columns were carried out as described previously [3,21,22]. MAb 3A5.4 was used for these studies due to its ability to react with the s/e NS1 glycoproteins of each DENV serotype, as well as the s/e NS1 glycoprotein of YFV. Briefly, highly concentrated stocks of MAb 3A5.4 were diluted in phosphate buffered saline (PBS) (pH 7.6) and slowly passed through a protein G-Sepharose (P3296, Sigma-Aldrich, USA) column, washed with PBS, eluted using 0.1 M glycine/HCl (pH 2.5), and 0.9-ml fractions were immediately neutralised with 100 μl of 2 M Tris/HCl (pH 7.8). High-MAb-containing fractions were identified using the bicinchoninic acid (BCA) protein assay (BCA-1, Sigma-Aldrich, USA), pooled and repeatedly dialysed against 2 litre volumes of 0.2 M NaHCO_3_/0.5 M NaCl (pH 8.9). Cyanogen-bromide-activated Sepharose 4B (0.8 g) (C9142, Sigma-Aldrich, USA) was pre-swollen and then washed with 500 ml of ice-cold HCl (1 mM). MAb 3A5.4 (12–15 mg) in 5 ml of the dialysate was then added, and the capped columns were incubated by inversion overnight at 4°C. The excess sites were then blocked with 1M ethanolamine/PBS/HCl (pH 9.0) for 2 h at ambient temperature. These columns were then subjected to nine cycles of high and low pH oscillations using 0.1 M glycine/HCl containing 1 M NaCl (pH 3.5) and 50 mM Tris/HCl containing 1 M NaCl (pH 9.0), before being washed with 10 mM diethylamine/PBS (pH 11.2) and then PBS containing 0.2% (wt/vol) NaN_3_ before being stored at 4°C.

### Growth of DENV strains of each DENV serotype and YFV and the immunoaffinity purification of their s/e NS1 glycoproteins

DENV strains of all four DENV serotypes were propagated in both insect (C6/36) cell and mammalian (Vero) cell monolayers as described previously [5,19]. For these studies, YFV (17D-204 vaccine strain) was also propagated in both C6/36 and Vero cells. High-titre seed stocks of each virus were prepared in 80 cm^2^ cultures of 70% confluent C6/36 cells maintained in Leibovitz L-15 medium (L4386, Sigma-Aldrich, USA) containing 10% (vol/vol) tryptose phosphate broth (T9157, Sigma-Aldrich, USA) with 10% (vol/vol) foetal calf serum (FCS), pyruvate, L-glutamine and antibiotics (referred to hereafter as insect cell growth medium (ICGM)). Cell culture supernatants were collected on days 4 and 8 after infection, clarified by centrifugation and stored at -80°C. These stocks were used to infect 70%-confluent mammalian (Vero) cell monolayers maintained in medium 199 containing Na_2_HCO_3_, 3.5% FCS, pyruvate, L-glutamine and antibiotics (referred to hereafter as mammalian cell growth medium (MCGM) in 10 large (225 cm^2^) flasks. After growth at 37°C in a 5% (vol/vol) CO_2_ atmosphere for 4 days, the supernatants were collected and replaced by fresh MCGM and incubated for a further 4 days before being harvested. These supernatants were made up to 20 mM Tris/HCl (pH 7.4), 1 mM PMSF, 5 mM Na_2_EDTA, 0.05% (wt/vol) NaN_3_, 7% (wt/vol) polyethylene glycol 8000 (89510, Sigma-Aldrich, USA) and 0.4 M NaCl using stock solutions. The DENV particles (virions) were then allowed to aggregate overnight at 4°C before being removed by centrifugation at 8,000 *× g* for 30 min at 4°C. The clarified supernatants were then slowly (1 ml/min) passed through the MAb 3A5.4 or 3D1.4 immunoaffinity columns. After washing with loading buffer, the bound extracellular/secreted (s/e) NS1 glycoproteins were eluted in their native homo-hexameric form using 20 mM diethylamine in 10 mM Tris/HCl containing 150 mM NaCl, PMSF and EDTA and 0.4 ml fractions were immediately neutralised with 100 μl of 1M Tris/HCl (pH 7.2). Protein concentrations were determined in ELISA plates using 10 μl of sample in 200 μl of BCA reagent (BCA-1, Sigma-Aldrich, USA) with standard concentrations (16 mg/ml to 125 μg/ml serial dilutions) of bovine serum albumin (A7906: Sigma, USA) concentrations prepared in neutralised elution buffer. ELISA plates were incubated at 37°C for 60 min and then absorbance was determined at 570 nm (MRX, Dynax, USA), and protein concentrations were derived from the standard curves. Fractions containing the DENV s/e NS1 glycoproteins were concentrated by centrifugation dialysis at 1,000–2,000 *× g* (Centricon 10, Amersham, UK) against RPMI-1640 medium (R6504, Sigma, USA) containing a cocktail of protease inhibitors (P1860, Sigma-Aldrich, USA). Protein concentrations were then determined again and fractions were stored at -80°C.

One 25 cm^2^ flask of DENV-2 infected Vero cells was also used to prepare an infected cell lysate for the Western blot assays by discarding the supernatant and repeatedly washing the cells with RPMI medium before the addition of 2 ml of 32 mM orthophosphoric acid/58 mM Tris base (pH 6.7) (345245/T6066: Sigma-Aldrich, USA) containing 10% sodium dodecyl sulphate (SDS) (L3771, Sigma, USA) (cell-lysis buffer). After repeated passage through a 23-gauge needle to break the DNA, the cell lysate was centrifuged at 200 *× g* and aliquots of the supernatant were stored at -80°C.

### Immunisation of mice and production of mouse MAbs

The immunisation and use of halothane in oxygen to anaesthetise BALB/c mice, and the production and cloning of MAbs were carried out as described previously [5,22,38]. Briefly, a group of three 6-week-old female BALB/c mice (Universidad Nacional, Bogota, Colombia) were immunised by the combined subcutaneous (s.c.; 0.1 ml) and intra-peritoneal (i.p.; 0.4 ml) routes with a mixture of 5 μg of the purified e/s NS1 glycoprotein of each DENV serotype (i.e., 20 μg/mouse) emulsified in complete Freund’s adjuvant (F5881, Sigma, USA). These mice were also each injected with 0.5 μg of mouse interleukin-12 (IL-12) by the i.p. route on days 1, 3, 5 and 7 after the immunisations to increase the production of PAbs of the IgG2a subclass [39]. Three weeks later, the mice were boosted with the sample antigen emulsified in incomplete Freund’s adjuvant (F5506: Sigma, USA) at the same dose and via the same route, and they also received the same multiple IL-12 injections. Four weeks later, the mice were anaesthetised and blood samples were obtained from the retro-orbital sinus. The highest responding mouse, as determined by the indirect ELISA titres against the s/e NS1 glycoprotein of each DENV serotype, was then immunised with 10 μg each of the DENV-1 (S#14 strain), DENV-2 (S#42 strain), DENV-3 (S#25 strain) and DENV-4 (S#10 strain) s/e NS1 glycoproteins (40 μg in total) in RPMI medium by the combined intravenous (i.v.) route in the tail vein (100 μl) and the i.p. route (300 μl). Three days later, this mouse was anesthetised and humanely killed by cervical dislocation, immersed in 70% ethanol and transferred to a class II laminar flow cabinet. The spleen cells were aseptically removed in RPMI medium containing 10% MAb-cloning tested quality FCS (FCS Premium: Biomeda, USA) and then centrifuged at 200 *× g* for 10 min at 28°C. SP2/0 Ag14 plasmacytoma cells (ATCC, USA), grown in RPMI medium containing 20% FCS and pyruvate, L-glutamine and antibiotics (PGAB), were harvested. The spleen and plasmacytoma cells were counted and mixed at a 10:1 ratio, co-centrifuged at 250 *× g* and the supernatant was discarded. One millilitre of 45% (wt/vol) polyethylene glycol 4000 (Merck BDH, UK) containing 5% dimethylsulfoxide (D2650, Sigma, USA)/PBS (pH 8.1) was slowly added to the loosened cell pellet over a period of 1 min with constant agitation before being slowly diluted with 20 ml of Hank’s balanced salt solution (H9394, Sigma, USA). After incubation at 28°C for 30 min, the cells were pelleted by centrifugation at 200 *× g*, the supernatant was discarded, and the cells re-suspended in 200 ml of RPMI medium containing 20% FBS, 1 × HAT (H0262: Sigma, USA) and PGAB before being added to 96-well culture plates (NUNC, USA) at 200 μl/well. After incubation at 37°C in 5% CO_2_/air for 5 days, 100 μl of the medium was changed and thereafter 150 μl was changed every 3 days until the DENV s/e NS1 glycoprotein MAb-positive wells were expanded. The 150 μl volumes collected from each well were tested by ELISA for reactions against the purified DENV s/e NS1 glycoprotein of each DENV serotype, and the contents of the wells containing MAbs that cross-reacted with the s/e NS1 glycoprotein of multiple DENV serotypes were transferred into 48-well plates (Costar, USA). HAT selection medium was subsequently substituted with medium containing HT (H0137, Sigma, USA) after 14 days, from which these hybridomas were never weaned. The culture supernatants were then tested for reactivity with the s/e NS1 glycoprotein from each DENV serotype in dot-blot assays. Western blot assays were also performed to test reactivity against a) the NS1 glycoproteins from each DENV serotype and YFV, b) the 2–mercaptoethanol reduced DENV-2 s/e NS1 glycoprotein, the cell-associated DENV-2 NS1 glycoprotein present in a crude SDS-lysate of DENV-2 infected Vero cells, and c) the DENV-2 s/e NS1 glycoprotein present in a human serum sample after the sera had been treated with 3% H_2_O_2_/PBS for 30 min to quench endogenous peroxidases. Thus, these assays were used to select wells containing hybridomas that secreted MAbs that specifically reacted with the s/e NS1 glycoprotein of each DENV serotype, and which would not cross-react with proteins in Vero cells or human sera.

Hybridomas that secreted the desired MAbs were cloned twice by limiting dilution in 96-well cell culture plates containing 2–4 × 10^4^/well CD1 (Swiss out-bred) mouse peritoneal macrophage feeder layers. Single wells were identified using an inverted microscopy, the supernatants were screened by ELISA against DENV s/e NS1 glycoproteins and the resultant cloned hybridomas were further sequentially expanded and harvested, pelleted by centrifugation and the cells slowly (-1°C/min) frozen to -80°C after being re-suspended in ice-cold 10% (vol/vol) DMSO/FCS, before being transferred to liquid nitrogen. From this fusion, MAb 2C4.6 and a number of other cloned hybridomas were selected.

The IgG subclass of the MAbs was determined by a kit using goat PAbs specific for each mouse IgG subclass in radial immunoassays (ISO-2, Sigma, USA), according the manufacturer’s instructions.

### Immunoassays

The indirect DENV s/e NS1 glycoprotein ELISA was performed as described previously [5,21,23]. Briefly, the immunoaffinity-purified DENV-2 s/e NS1 glycoproteins (see above) or the negative-control antigen, bovine serum albumin, were diluted to 3 μg/ml in carbonate/bicarbonate buffer (pH 9.8), loaded into 96-well ELISA plates (Immulon 2, Dynatech, USA) and adsorbed overnight at 4°C. After washing with PBS containing 0.05% (vol/vol) Tween20 (PBS/T), blocking with 1% (wt/vol) gelatin/PBS for 2 h at 25°C and washing again with PBS/T, 50 μl volumes of the undiluted hybridoma wells were transferred to each ELISA well and allowed to react at 37°C for 60 min. After washing with PBS/T, the bound MAbs were detected with sequential reaction steps using peroxidase-labelled goat anti-mouse IgG heavy and light (H&L) chain reactive (115-035-166, Jackson ImmunoResearch, USA) in PBS/T/G, washing with PBS/T, incubation with *o*-phenylenediamine (opd) in citrate/phosphate buffer (pH 5.0) containing H_2_O_2_ and the absorbance values measured at 490 nm (MRX: Dynex, USA).

The Western blot assays were performed as described previously [3,21,22]. Samples were prepared using 1.0 μg s/e NS1 glycoprotein of each DENV serotype added to 160 μl of stacking buffer containing 0.5% (wt/vol) SDS, 32 μl of 0.25% (wt/vol) bromophenol blue in 50% glycerol/H_2_O, with (all DENV serotypes) or without (DENV-2 s/e NS1 glycoprotein) 1% (vol/vol) 2-mercaptoethanol (M3148: Sigma, USA). In addition, 160 μl of a DENV-2 infected Vero cell lysate (see cell culture) and 30 μl of a normal human serum sample (which had been diluted 1 in 4 with PBS) were both treated with 3% (vol/vol) H_2_O_2_ (H1009, Sigma, USA) for 60 min at 28°C to quench endogenous human serum peroxidases, before the addition of the 5 × stacking buffer and 1.0 μg of DENV-2 s/e NS1 glycoprotein (total volume 160 μl). Both were then treated with 32 μl of 0.05% (wt/vol) bromophenol blue in 50% glycerol/H_2_0. These samples were then heated at 100°C for 3 min. After cooling and centrifugation, they were then loaded onto 8–9% (wt/vol) preparative SDS/PAGE gels (Miniprotean II: Bio-Rad, UK) and subjected to electrophoresis (15–20 constant mA/gel) and subsequent electro-blotting at 160 mA/gel (Sartoblot II, Sartorius, UK) onto 0.2-μm pore-sized nitrocellulose membranes (BioTraceNT, PAL LifeSciences, USA) before being dried. These membranes were then blocked using 3% (wt/vol) skimmed milk powder (Marvel, Cadbury, UK)/PBS (PBS/T/M) containing 0.02% (wt/vol) NaN_3_, washed with PBS/T, dried and then cut into 3-mm wide strips. Preparative blot strips of the reduced (DENV-2 s/e NS1 glycoprotein only) and non-reduced (s/e NS1 glycoproteins of all DENV serotypes and YFV, DENV-2 infected Vero cells and human sera which contained the DENV-2 s/e NS1 glycoprotein) were taped together with waterproof autoclave tape and allowed to react with 0.5 ml of the MAb supernatants of two 500 μl samples (1 ml total) from 48-well cultures diluted with an equal volume of PBS/T containing 2% (wt/vol) skimmed milk powder for 2 h at ambient temperature. After washing with PBS/T, the bound MAbs were detected using sequential reaction steps with peroxidase-labelled goat anti-mouse IgG (H&L) (115-035-166: Jackson ImmunoResearch, USA) in PBS/T/G, washing with PBS/T, PBS and then reacting the membranes with 4-chloro-1-naphthol/3,3’diaminobenzidine (C8890/D8001, Sigma, USA) (CND) substrate in PBS containing 0.006% (vol/vol) H_2_O_2_.

For the dot-blot assays, 0.5-cm squares were marked on the nitrocellulose membranes using an 8B graphite pencil (Staedtler, Germany). Duplicate, serial, two-fold or three-fold dilutions of the immunoaffinity-purified s/e NS1 glycoproteins prepared in normal human sera collected from anti-DENV IgM and IgG-negative patients [19] were added at 10 μl/dot and were air dried. Different methods were assessed to quench the endogenous peroxidases in the human sera present on these dot-blots. For this study, solutions of 3% H_2_O_2_ diluted from a 30% (vol/vol) stock solution (H1009, Sigma Aldrich, USA) in 40% (vol/vol) MeOH/PBS, PBS or H_2_O were tested. These dot-blot assays were then processed as described for the Western blot assays to identify the detection sensitivities of MAb 2C4.6 against the s/e NS1 glycoprotein of each DENV serotype when reacted at a concentration of 5–10 μg/ml with serial dilutions of each of them. The sensitivities of MAb 2C4.6 and 3D1.4 against known concentrations of the s/e NS1 glycoprotein of each DENV serotype were then compared. The reactions of other MAbs anti-NS1 MAbs (1G5.4-A1-C3 and 1B6.3) of the IgG2b subclass, which defined a DENV-2 > DENV-4 > DENV-1 > DENV-3 and DENV-2-specific responses against the s/e NS1 glycoprotein of each DENV serotype in Western blot assays respectively [3,21,22], were also tested for their reactions in these dot-blot assays.

In addition, we assessed whether: a) 1 mM Ni^2+^ (NiCl_2_) could increase the sensitivities of these assays when added to the 4-chloro-1-naphthol/3,3’ diaminobenzidine (CND) substrate mixture; b) the assays could be successfully processed without loss of detection sensitivities after sample addition and blocking, washing with PBS containing 0.01% NaN_3_ and storing after drying for 1 month at 28°C (ambient temperature), 4°C, -20°C and -80°C; and c) the total assay time could be reduced to 3 h without significant loss of detection sensitivity when they were performed at ambient temperature.

## Competing interests

The authors declare that they have no competing interests.

## Authors’ contributions

The authors both contributed to the design of the experiments, the laboratory work and preparation of the article. Both authors read and approved the final manuscript.
